# Is There a Need for Abdominal CT Scan in Trauma Patients With a Low-Risk Mechanism of Injury and Normal Vital Signs?

**DOI:** 10.7759/cureus.11628

**Published:** 2020-11-22

**Authors:** David Ledrick, Alexander Payvandi, Adam C Murray, John J Leskovan

**Affiliations:** 1 Department of Emergency Medicine, Mercy St. Vincent Medical Center, Toledo, USA; 2 Department of Trauma Surgery, Mercy St. Vincent Medical Center, Toledo, USA

**Keywords:** trauma centers, lung injury, retrospective studies, x-ray computed tomography, non-penetrating wounds

## Abstract

Background

Clinically significant injuries are often missed in trauma patients with low-risk mechanisms of injury and lack of "red flags," such as abnormal vital signs. The purpose of this retrospective analysis was to evaluate the efficacy of computed axial tomography (CT) for identifying occult injuries in a high-volume trauma center.

Methods

Records from our institutional trauma registry were retrospectively extracted, examining encounters from January 2015 to October 2019. Those patients between the ages of 18 and 65 who were referred to the trauma team with a CT scan of the abdomen and had low-risk mechanisms of injury, a Glasgow Coma Scale (GCS) score of 15, and normal vital signs at presentation were included. Patients in the lowest trauma categorization (Level Three, Consult) met the study definition for the low-risk mechanism of injury. Demographic and clinical data were abstracted for all patients. For this analysis, patients were divided into two groups based on age (18 - 40 years or 40 - 65 years). Injuries found on CT, their clinical significance, and the likelihood of being missed without CT were determined.

Results

Of 2,103 blunt trauma patients that received a CT scan of the abdomen from January 2015 to October 2019, 134/2,103 (6.4%) met the inclusion criteria (mean age: 44.6 years; 72.3% male). Patients between the ages of 40 and 65 years comprised 61.2% (82/134) of the study population. Of the included patients, 17.2% (23/134) had at least one acute traumatic injury identified after CT imaging of the torso. Occult injuries found on CT included rib fracture with associated lung injuries (10/23, 43.5%), splenic laceration (4/23, 17.4%), liver laceration (3/23, 13.0%), gluteal hematoma with active bleeding (1/23, 4.3%), sternal fractures (3/23, 13.0%), and thoracic or lumbar spine fractures (2/23, 8.7%). An independent review of the medical records determined that 9.0% (12/134) of these patients had traumatic injuries that would have been missed based on clinical examination without CT.

Conclusions

Based on our experience, utilizing CT imaging of at least the abdomen as a routine screening measure for all trauma consults - even low-risk patients with normal vital signs - can rapidly and accurately identify clinically significant injuries that would have been otherwise missed in a notable portion of the population.

## Introduction

Rapidly identifying and initiating treatment for life-threatening injuries can improve clinical outcomes for trauma patients; however, such clinically significant injuries are often overlooked in the presence of more obvious external injuries, resulting in delayed diagnoses and contributing to trauma mortality [1-2]. Computed axial tomography (CT) can provide sensitive and specific evaluations of trauma patient injuries [3-8]. Innovations in the current generation of CT systems enable improvements in the speed, accuracy, quality, and availability of such injury evaluations [9]. Earlier CT models were limited by their length of scanning time and location of equipment, which could be particularly challenging for unstable trauma patients. Newer iterations of CT technology offer higher slice models that can increase area coverage and produce sharper images, allowing many institutions to perform scans within minutes in highly monitored emergency departments (ED) or trauma bays [9-12].

The integration of CT scanning into emergency settings can be particularly useful for trauma patients with high-risk occult injuries that may be otherwise missed, such as solid organ damage, hemothorax, pneumothorax, lung contusions, thoracic injuries, post-traumatic atelectasis, and unsuspected fractures of the pelvis or spine [1-2, 8, 12-14]. Uniform CT scanning for trauma patients can reduce the rates of missed injuries resulting from an inadequate examination, reducing the rates of avoidable mortality. For these reasons, it may be beneficial to utilize CT imaging as a screening tool in all trauma patients, including those considered to have normal physical examinations [3-8, 12].

In order to evaluate the benefit of routine CT's, we examined the rate of injuries in a population of adult trauma patients presenting with normal vital signs and low-risk mechanism of injury. We made a further effort at determining the clinical significance of these injuries and whether they would have likely been missed without CT.

## Materials and methods

This study was approved by the local Institutional Review Board (IRB) of Mercy St. Vincent Medical Center (approval #2020-10-MHSVMC). Informed consent was waived due to the retrospective nature of this study. Adult trauma patients were retrospectively identified from the institutional trauma registry from January 2015 through October 2019. Pediatric patients (< 18 years), geriatric patients (> 65 years), and patients with unavailable data were excluded from our analysis.

Inclusion criteria

**Figure 1:**
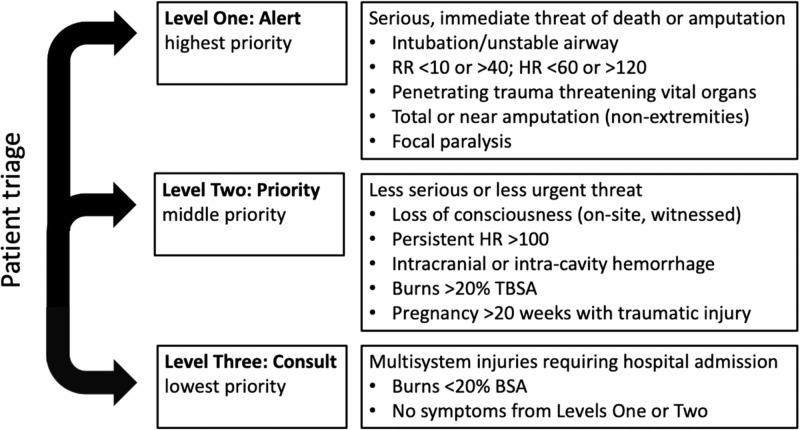
Criteria for categorization of patients based on trauma level at presentation This criterion for trauma triage is based on the American College of Surgeons’ *Resources for Optimal Care of the Injured Patient *and adapted based on our institutional experience [15]. The receiving staff categorizes the patient based on their highest level of symptoms so that an appropriate team is available for care; patients lacking any Level One or Level Two symptoms are triaged to Level Three. BSA: body surface area; HR: heart rate; RR: respiration rate; TBSA, total body surface area

Patients referred to the trauma team that had at least a CT scan of the abdomen performed, low-risk mechanisms of injury, and normal vital signs at presentation were included. The low-risk mechanism of injury was defined using trauma categorization as per our institutional protocols for labeling a trauma: alert, priority, or consult (Figure 1). These evaluations are performed by the trauma team upon arrival at the ED. Level One Trauma, or trauma alert, refers to patients with unstable airways, significantly abnormal vital signs, significant interventions required in the field, and the anticipated need for immediate, life-saving intervention. Level Two Trauma, or trauma priorities, are patients at risk of developing Level One Traumas based on medical history, risk factors, or physical findings but have not yet decompensated. Level Three Trauma, or trauma consults, are those who do not meet any of the above criteria but are still at risk for an occult injury. By definition, only those in the Level Three Trauma classification met the low-risk mechanism of injury criteria. Normal vital signs were defined as patients with all three of the following criteria: a pulse < 90, a systolic blood pressure > 110 mm Hg, and a Glasgow Coma Scale (GCS) of 15. 

Data and analysis

Demographic data and clinical information were abstracted for each patient. Initial vital signs (i.e., pulse, blood pressure, GCS), mechanism of injury, and results of the chest x-ray and CT scans by body region were also recorded. If traumatic findings were identified, medical records were reviewed to determine if a change in management was initiated. The CT scan results and subsequent management were recorded and reviewed by a trauma surgeon or emergency medicine physician. Patients were also categorized by age for our analysis, with one group comprised of patients 18 - 40 years old, and a second group comprised of patients 40 - 65 years old.

## Results

Demographic data

The trauma registry had complete data on 2,103 blunt trauma patients between the ages of 18 and 65 who received at least a CT scan of the abdomen from January 2015 to October 2019. There were 523 (24.9%) Level One Trauma patients, 1,097 (52.1%) Level Two Trauma patients, and 420 (20.0%) Level Three Trauma patients identified; 63 (3.0%) unclassified patients were excluded. Of the total patients identified, 134 (6.4%) met all the following inclusion criteria of the low-risk mechanism of injury, normal vital signs on initial presentation, and a GCS of 15. Patients over the age of 40 comprised 61.2% (82/134) of the included population. The study population was 72.3% (97/134) male, with a mean age of 44.6 years.

Acute traumatic injuries

Of the study population, 17.2% (23/134) had at least one acute traumatic injury identified by CT imaging of the torso (Table 1). The injury most frequently identified was rib fracture with associated lung injuries (10/23, 43.5%). Four patients with splenic lacerations (4/23, 17.4%) presented with Grades I, III, III, and IV on the American Association for the Surgery of Trauma (AAST) splenic injury scale [16]. Three patients with liver lacerations (3/23, 13.0%) presented with grades I, III, and IV on the AAST liver injury scale. Three patients (3/23, 13.0%) suffered from Grade I, II, and III renal injuries on the AAST renal injury scale [16]. Sternal fractures (3/23, 13%) and thoracic or lumbar spine fractures (2/23, 8.7%) were also observed. One patient (1/23, 4.3%) with a gluteal hematoma suffered active bleeding. Age did not have a notable effect on the rate of injury; patients under the age of 40 (52/134, 38.8%) accounted for 40.0% (4/10) of the solid organ injuries, including two of the liver lacerations (2/3, 66.6%), one of the splenic lacerations (1/4, 25.0%), and one of the renal lacerations (1/3, 33.3%).

**Table 1:** Acute Injuries Identified After CT Scanning

Traumatic Injury	Number of Patients
Rib fracture with associated lung injury	10
Splenic laceration	4
Liver laceration	3
Sternal fracture	3
Renal injury	3
Thoracic or lumbar spine fracture	2
Gluteal hematoma	1

Clinical significance

An independent emergency medicine physician with 20 years of experience reviewed the medical charts and determined that these traumatic injuries would have been missed in 9.0% (12/134) of the total included patients, as the traumatic findings were not identified by plain films, laboratory abnormalities, or ultrasound imaging. Management was altered in all 23 of the patients with acute traumatic injuries identified by CT scans; all were admitted and monitored, 8.7% (2/23) required a splenectomy, 4.3% (1/23) was sent to interventional radiology for splenic embolization, and 8.7% (2/23) underwent neurosurgical procedures. Of the remaining patients, the CT scan provided a better resolution of the injury than clinical evaluation alone but was not considered essential in changing the outcomes. 

## Discussion

Our retrospective analysis demonstrated that CT scanning of low-risk trauma patients with normal vital signs identified additional acute injuries that would have otherwise been missed. These findings allowed the courses of treatment to be appropriately modified and additional interventions to be initiated. Given the low-risk categorization upon presentation, these findings suggest that introducing CT as a screening measure in trauma patients may prove beneficial for the evaluation of coexisting and unsuspected injuries. Each institution should continuously evaluate their accepted systems of patient care to maximize resources, efficiency, and positive clinical outcomes.

The topic of CT efficacy for the evaluation of injuries in trauma patients is highly debated in the literature [3, 6-8, 17-19]. Although several efforts have previously been made to implement judicious use of diagnostic algorithms and CT imaging in emergency and trauma settings [18-23], indiscriminate CT imaging as a screening measure, especially for low impact injuries, may pose a challenge for already strained healthcare infrastructures [17, 24-25]. Uniform CT scans in trauma patients with normal vitals may delay diagnosis and treatment for other patients with non-traumatic but serious illnesses. Furthermore, introducing whole-body CT systems has previously been associated with higher doses of radiation on average, which may contribute to an accumulated lifetime risk of malignancy in both adult and pediatric patients [18, 26-28].

Despite the potential challenges associated with introducing CT imaging as a routine diagnostic tool for all trauma consults, our experience indicates the benefit of using CT to rapidly and accurately evaluate occult injuries in order to institute appropriate treatment decisions. Similarly, Tillou et al. assessed the efficacy of CT scanning in a population of 94 low-risk trauma patients [5]. They found that significant injuries were identified in 17% of the scans that would have been missed otherwise, and injuries requiring immediate intervention were identified in two scans [5]. Nellensteijn et al. also investigated CT imaging in 64 hemodynamically stable children with blunt trauma; of these patients, 6% died and CT brought forward an indication for intervention in 5% of patients [28]. Additionally, James et al. demonstrated a reduction in missed diagnoses with a routine pan-scanning protocol for trauma assessment in a prospective analysis [3]. During this study, missed injuries decreased from 3.2% to 0.5%, and incidental findings increased from 8% to 14% with the implementation of routine pan-scans [3]. Based on these findings, and our own institutional experiences, we believe routine CT can increase diagnostic confidence and reduce the incidence of complications or deaths associated with missed injuries. 

The economic burden to both the patients and the institutions may limit the widespread adoption of CT scanning in the radiographic workups of all trauma patients [3, 27, 29]. A micro-cost analysis estimates the cost of routine CT screening to be $4,029 per blunt trauma patient [30]. Although whole-body scanning has higher direct costs and delivers higher doses of radiation on average, routine pan-scanning can significantly accelerate time to diagnosis and intervention, reducing both the length of stay in an ED and the need for additional CT examinations [3, 7, 26]. Thus, while uniformly screening trauma patients with CT might result in a higher initial cost, these measures may reduce operational costs elsewhere. One study evaluating the direct costs of scanning estimated that 38% (416/1,097) of the scans at their center were reflexively ordered for malpractice defensive purposes only [29]. These efforts resulted in $120,000 of additional charges, but significant injuries were discovered in 2.2% that would have gone undiagnosed [29].

The present investigation has several important limitations, including its retrospective design. There was no investigation into trauma level misclassification as it was felt that the trauma team at the time had a better perspective on mechanism than any retrospective chart reviewer; however, the possibility of misclassification remains. No effort was made to include trauma patients from the ED who were treated and discharged without trauma team involvement, as these patients were not documented in the registry and there was no clear mechanism for identifying them retrospectively. Furthermore, only a small portion of trauma patients from our institution were included due to the strict inclusion criteria of absolutely normal vital signs, which limits the size of the cohort and generalizability of these findings. A larger prospective study following the course of each patient presenting with a low-risk mechanism of injury would provide a more complete set of data. Future studies should also investigate the efficacy of routine CT scanning for identifying unsuspected injuries in a population of higher-risk trauma patients.

## Conclusions

Rapid identification of occult injuries in trauma patients remains a challenge in all trauma centers. Based on our experience with low-risk trauma patients with normal vital signs, introducing routine CT imaging of at least the abdomen can facilitate diligent and timely screening for patients referred to a trauma team for evaluation, reduce the number of missed injuries, and result in the initiation of appropriate interventions. Barriers, such as cost, may affect the more widespread adoption of routine CT scanning for trauma patients.
